# Assessing the accuracy of the updated Fujifilm SILVAMP TB LAM II assay between lot numbers

**DOI:** 10.5588/ijtldopen.24.0449

**Published:** 2025-02-01

**Authors:** K. Chikamatsu, Y. Shimomura, A. Osugi, S. Mitarai

**Affiliations:** ^1^ Bacteriology Division, Department of Mycobacterium Reference and Research, The Research Institute of Tuberculosis, Japan Anti-Tuberculosis Association, Tokyo, Japan;; ^2^ Basic Mycobacteriosis, Nagasaki University Graduate School of Biomedical Sciences, Nagasaki, Japan.

**Keywords:** FujiLAM II, urine-based lipoarabinomannan, lot-to-lot variability, people living with HIV

## Abstract

**BACKGROUND:**

The Fujifilm SILVAMP TB LAM (FujiLAM) assay, a point-of-care test detecting urine-based lipoarabinomannans, has demonstrated notable diagnostic accuracy for TB. However, FujiLAM exhibits lot-to-lot variability, limiting its clinical use. A new version of the product, Fujifilm SILVAMP TB LAM II (FujiLAM II), was developed to address this issue. This study evaluated the lot-to-lot variability and diagnostic accuracy of the FujiLAM II.

**METHODS:**

We assessed three lots of FujiLAM II using 158 biobanked, frozen urine specimens from people living with HIV (PLHIV): 72 TB-positive and 86 TB-negative, according to the microbiological reference standard.

**RESULTS:**

Independent proportions of the results among the three FujiLAM II lots did not differ (Cochrane *Q* test: *P* > 0.37). Overall, sensitivities of the three FujiLAM II lots were 78.3%, 79.7% and 79.7%, and specificities were 92.9%, 93.0% and 93.0%, respectively. In patients with CD4 cell counts <200 cells/µL, sensitivities of the three FujiLAM II lots were 97.8%, 100% and 100%, and specificities were 91.7%, 91.8% and 91.8%, respectively.

**CONCLUSION:**

The FujiLAM II demonstrated no lot-to-lot variability and exhibited high sensitivity and specificity for TB diagnosis in patients with CD4 cell counts <200 cells/µL. The FujiLAM II enhanced reproducibility as a TB diagnostic tool in PLHIV.

TB is estimated to claim 1.3 million lives annually worldwide.^1^ Likewise, approximately 6.63 million HIV-related deaths are recorded globally every year, 167,000 of which are attributable to TB.^1^ *Mycobacterium tuberculosis* detection typically requires sputum; however, people living with HIV (PLHIV) who have severe TB have difficulty producing sputum. Furthermore, cases of extrapulmonary TB are more common in PLHIV than in patients without immunodeficiency.^2^ Therefore, standard bacteriological tests for TB have limited diagnostic capacity in PLHIV. Consequently, there is a need for TB diagnosis using specimens other than sputum.

Fujifilm SILVAMP TB LAM (FujiLAM; Fujifilm, Tokyo, Japan), which was developed as a lateral flow test to detect lipoarabinomannan (LAM) antigen (a mycobacterial cell wall component) in urine, emerged as a promising solution for TB detection. The FujiLAM showed superior sensitivity for TB diagnosis in biobanked, frozen urine specimens from PLHIV compared to Alere Determine TB LAM Ag Assay (AlereLAM; Abbot, Chicago, IL, USA): 70.7% vs. 34.9%, respectively.^3^ However, variations in sensitivity and specificity have been primarily attributed to lot-to-lot differences in accuracy, rather than country-specific factors, raising concerns about the consistency of the assay and limiting its clinical application.^4,5^

The manufacturing process was modified to address this issue, and a new version of the product Fujifilm SILVAMP TB LAM II (FujiLAM II) was developed. This study aimed to evaluate the lot-to-lot variability and diagnostic accuracy of FujiLAM II using urine specimens from PLHIV.

## METHODS

### Clinical specimens

A total of 158 PLHIV urine specimens from the following regions: South Africa: 38, Malawi: 51, Zambia: 35, Tanzania: 18, Vietnam: 9, Thailand: 7, were obtained from the Foundation for Innovative Diagnostics (FIND; Geneva, Switzerland) Specimen Bank. Urine specimens were collected between December 2019 and July 2021 as part of a study facilitated by FIND. The exact storage dates are not specified, but we assume the samples were stored immediately after collection, as indicated by FIND. Urine specimens were stored at –80°C and thawed at 4°C at the time of use. Thawed urine specimens were brought to room temperature before testing and mixed by inversion. A microbiological reference standard (MRS) was used to assess the TB diagnostic accuracy of the FujiLAM II. MRS was defined as positive for at least one of the following microbiological tests: TB culture (Mycobacteria Growth Indicator Tube [MGIT; BD, Franklin Lakes, NJ, USA], Löwenstein-Jensen, blood culture) and Xpert^®^ MTB/RIF Ultra (Cepheid, Sunnyvale, CA, USA) of sputum and urine. MRS was considered negative when none of these tests returned a positive result, and at least one test, such as Ultra of urine or other microbiological test, was confirmed negative.^6^ The MRS data and CD4 cell counts were obtained from FIND. Of the 158 specimens, 13 were previously evaluated with six lots of FujiLAM in the original study, and an additional lot was included in the current evaluation, all of which were TB-negative according to MRS.^5^

### FujiLAM II and AlereLAM assays

The FujiLAM II and AlereLAM assays were performed according to the manufacturer’s instructions (Figure). Three FujiLAM II lots (Lot 91000005, Lot 11300005 and Lot 11300007) and AlereLAM were tested simultaneously with the same urine specimens from the Research Institute of Tuberculosis, Japan Anti-Tuberculosis Association (RIT/JATA, Tokyo, Japan). Test results were independently read by two readers blinded to the TB diagnosis. When the two results disagreed, the readers discussed them and reached a consensus. The readers of each FujiLAM II lot were also blinded to the results of the other lots and to AlereLAM. This ensured independent interpretation of each set of results, avoiding bias and cross-referencing during simultaneous testing with the same urine specimens. If the test results were invalid, the test was repeated on the same day. Each FujiLAM II used in this study was produced from a different production run. The components, such as the labelled antibodies and the membrane coated with detection antibodies, were sourced from different processing batches, following standard industry practices.

**Figure.**
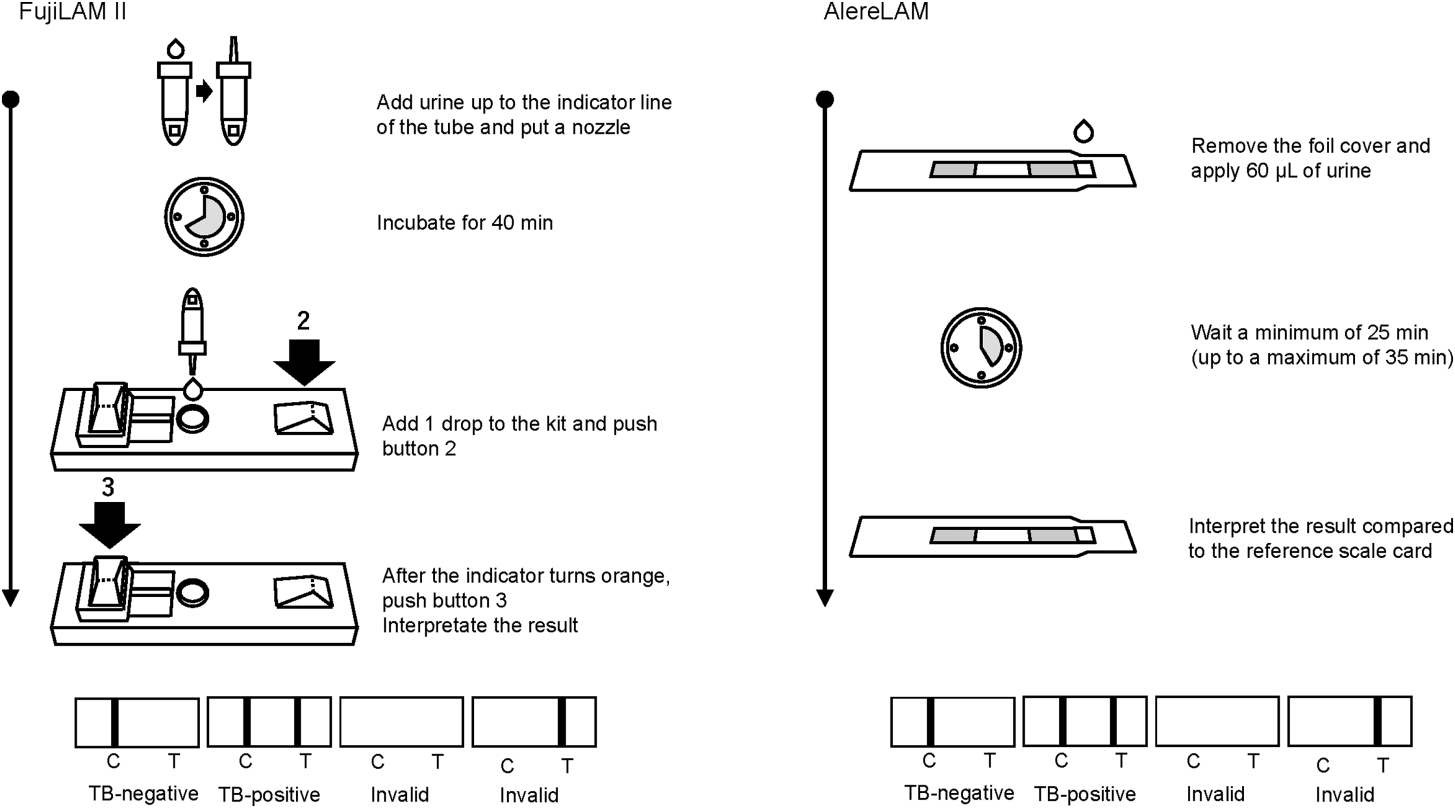
FujiLAM II and AlereLAM assay testing procedures. LAM = lipoarabinomannan; FujiLAM II = Fujifilm SILVAMP TB-LAM II; AlereLAM = Alere Determine TB LAM Ag.

### Statistical analysis

Urine specimens with invalid test results on retest were excluded from relevant analyses. The results from the three FujiLAM II lots and AlereLAM tests were compared with the MRS-TB diagnosis and classified as true-positive, false-negative, true-negative, or false-negative; sensitivity and specificity were also determined. Additionally, patients were classified according to CD4 cell counts, and the sensitivity and specificity were determined for each group. Cochran’s *Q* test was used to compare the independent proportions of FujiLAM II, and McNemar’s test was used to compare the sensitivity and specificity of FujiLAM II and AlereLAM. Similarly, Cochran’s *Q* and McNemar’s tests were used to compare the independent proportions and specificities of the seven lots of FujiLAM and three lots of FujiLAM II. Data were analysed using R v4.1.2 (R Foundation for Statistical Computing, Vienna, Austria). Statistical significance was set at *P* < 0.05.

### Ethical statement

Ethical approval was not required for this laboratory-based study, which used only urine specimens from a specimen bank. This study was approved by the Institutional Review Board of the RIT/JATA (#30-16). The FujiLAM II were provided by Fujifilm Corporation. The manufacturer had no involvement in the study’s design, conduct, analysis, or writing of the manuscript of this study.

## RESULTS

Of the 158 specimens, 72 were TB-positive, and 86 were TB-negative, according to MRS classification. Six cases could not be tested with AlereLAM because of insufficient specimen volume. FujiLAM II Lot 91000005, Lot 11300005 and Lot 11300007 were judged invalid in four, three and three cases, respectively, because the controls showed no colour. Urine specimens were retested and yielded consistent results. No AlereLAM was considered invalid. Table 1 presents the results of the study. Overall, there was no difference in the independent proportions of the decision results for the three FujiLAM II lots (Cochrane’s *Q* test: *P* > 0.37). The sensitivities of the three FujiLAM II lots (Lot 9100005, Lot 1130005 and Lot 1130007) were respectively 78.3% (95% confidence interval [CI] 68.5–88.0), 79.7% (95% CI 70.2–89.2) and 79.7% (95% CI 70.2–89.2). The sensitivity of AlereLAM was 54.9% (95% CI 43.4%–66.5), which was significantly lower than that of FujiLAM II (McNemar’s test: *P* < 0.0001). The specificities of the three FujiLAM II lots (Lot 91000005, Lot 11300005 and Lot 11300007) were respectively 92.9% (95% CI 87.5–98.4), 93.0% (95% CI 87.6–98.4) and 93.0% (95% CI 87.6–98.4). The specificity of AlereLAM was 92.6% (95% CI 86.9–98.3), which did not differ significantly from that of FujiLAM II (McNemar’s test: *P* = 0.71).

**Table 1.** Sensitivity and specificity of FujiLAM II and AlereLAM for microbiological reference standards.

Lot no.	TP	FP	FN	TN	Invalid	Sensitivity % (95% CI)	Specificity % (95% CI)
FujiLAM II							
All specimens							
91000005	54	6	15	79	4	78.3 (68.5–88.0)	92.9 (87.5–98.4)
11300005	55	6	14	80	3	79.7 (70.2–89.2)	93.0 (87.6–98.4)
11300007	55	6	14	80	3	79.7 (70.2–89.2)	93.0 (87.6–98.4)
CD4 <200 cells/μL							
91000005	45	5	1	55	4	97.8 (93.6–100)	91.7 (84.7–98.7)
11300005	46	5	0	56	3	100.0 (100–100)	91.8 (84.9–98.7)
11300007	46	5	0	56	3	100.0 (100–100)	91.8 (84.9–98.7)
CD4 ≥200 cells/μL							
91000005	9	1	14	24	0	39.1 (19.2–59.1)	96.0 (88.3–100)
11300005	9	1	14	24	0	39.1 (19.2–59.1)	96.0 (88.3–100)
11300007	9	1	14	24	0	39.1 (19.2–59.1)	96.0 (88.3–100)
AlereLAM							
All samples	39	6	32	75	6	54.9 (43.4–66.5)	92.6 (86.9–98.3)
CD4 <200 cells/μL	36	5	12	52	5	75.0 (62.8–87.3)	91.2 (83.9–98.6)
CD4 ≥200 cells/μL	3	1	20	23	1	13.0 (0–26.8)	95.8 (87.8–100)

LAM = lipoarabinomannan; FujiLAM II = Fujifilm SILVAMP TB-LAM II; AlereLAM = Alere Determine TB LAM Ag; CI = confidence interval; TP = true-positive; FP = false-positive; FN = false-negative; TN = true-negative.

In patients with CD4 cell counts <200 cells/µL, there was no difference in the independent proportions of decision results among the three lots of FujiLAM II (Cochrane’s *Q* test: *P* > 0.37). The sensitivities of the three FujiLAM II lots (Lot 91000005, Lot 11300005 and Lot 11300007) were respectively 97.8% (95% CI 93.6–100), 100% and 100%. The sensitivity of AlereLAM was 75.0% (95% CI 62.8–87.3), significantly lower than FujiLAM II’s (McNemar’s test: *P* = 0.0027). Specificities of the three FujiLAM II lots (Lot 91000005, Lot 11300005 and Lot 11300007) were respectively 91.7% (95% CI 84.7–98.7), 91.8% (95% CI 84.9–98.7) and 91.8% (95% CI 84.9–98.7). The specificity of AlereLAM was 91.2% (95% CI 83.9–98.6), which did not differ significantly from that of FujiLAM II (McNemar’s test: *P* = 0.71).

In patients with CD4 cell counts ≥200 cells/µL, the three lots of FujiLAM II had matching ratios of results by the MRS. The sensitivity of the three FujiLAM II lots (Lot 91000005, Lot 11300005 and Lot 11300007) was 39.1% (95% CI 19.2–59.1). The sensitivity of AlereLAM was 13.0% (95% CI 0–26.8), significantly lower than FujiLAM II’s (McNemar’s test, *P* = 0.014). The specificity of all three FujiLAM II lots (Lot 91000005, Lot 11300005, and Lot 11300007) was 96.0% (95% CI 88.3–100). The specificity of AlereLAM was 95.8% (95% CI 87.8–100), which did not differ from that of FujiLAM II.

In 13 specimens, there was a significant difference in the independent proportions of the decision results among the 10 lots (seven lots of FujiLAM and three lots of FujiLAM II) (Cochrane’s *Q* test: *P* = 3.02E^-10^). Specificities of the seven lots of FujiLAM ranged from 23.1–100%, whereas the three lots of FujiLAM II demonstrated a specificity of 100% (Table 2). Specificities of the four lots of FujiLAM (Lot 19001, Lot 19002, Lot 19003, and Lot 21001) were significantly less than that of the three FujiLAM II lots (McNemar’s test: *P* = 0.014–0.05).

**Table 2.** Comparisons between FujiLAM and FujiLAM II using 13 MRS-negative urine specimens.

	FujiLAM lot numbers							FujiLAM II lot numbers		
	19001	19002	19003	20002	20003	20004	21001	91000005	11300005	11300007
TN	3	5	5	10	13	13	8	13	13	13
FP	10	8	8	3	0	0	5	0	0	0
Positive rate, %	76.9	61.5	61.5	23.1	0	0	38.5	0	0	0
Specificity, %^‡^	23.1*	38.5*	38.5*	76.9	100	100	61.5^†^	100	100	100

* *P* = 0.014.

† *P* = 0.047.

‡ The specificity of seven lots of FujiLAM was compared with that of FujiLAM II using McNemar's test.

LAM = lipoarabinomannan; FujiLAM II = Fujifilm SILVAMP TB-LAM II; AlereLAM = Alere Determine TB LAM Ag; MRS = microbiological reference standard; TN = true-negative; FP = false-positive.

## DISCUSSION

Previous studies have evaluated the diagnostic accuracy of FujiLAM and AlereLAM point-of-care TB diagnostics, primarily focusing on PLHIV in multicentre settings.^3,4,7^ FujiLAM was assessed using various TB diagnostic criteria, including MRS, extended microbiological reference standard (eMRS), clinical reference standard (CRS), and HIV severity, as determined by the CD4 cell count. However, the diagnostic accuracy of FujiLAM for TB varies across studies. This variation was suspected to be due to the differences between lots, geographic locations, and visit days. High positivity rates are associated with specific lots, and this variation is particularly pronounced in patients with higher CD4 cell counts.^4^ Székely et al. reported positivity rates of 13–77% for different lots using the same specimens.^5^ Consequently, the FujiLAM was modified by the manufacturer to eliminate lot-to-lot variability. In this study, all lots were evaluated using the same specimens in a single laboratory by the same readers to ensure consistency. The FujiLAM II demonstrated no lot-to-lot variability across all specimens, including those stratified with the CD4 cell count. The sensitivity and specificity of FujiLAM were reported to be 60–77% and 87–93%, respectively, for diagnosing TB in PLHIV, while the diagnostic accuracy of the FujiLAM II was comparable to or better than those previously reported.^3,4,7–10^ Furthermore, we compared seven lots of FujiLAM with three lots of FujiLAM II using 13 specimens with a negative TB diagnosis, according to MRS. False positives in the FujiLAM were not observed in the FujiLAM II.

FujiLAM employs a lateral flow test using a sandwich immunoassay that has been used to measure proteins, such as enzymes, antibodies, cytokines, and hormones. Immunoassays offer high sensitivity and specificity; however, maintaining the quality of materials used in immunoassays, such as solid phases, antibodies, and labelling agents, is more challenging than chemical assays, making lot-to-lot variability a potential issue.^11^ Clinically, significant lot-to-lot variability may pose a risk to patient care.^12^ Although manufacturers implement internal quality control procedures, these may not be sufficient to detect significant changes in reagent performance.^13^ Therefore, a robust quality management system is essential for clinical use. To ensure test accuracy, FujiLAM II and other newly developed clinical products must be continuously monitored by measuring quality control materials that closely resemble clinical specimens.

This study had some limitations. First, we evaluated three lots of FujiLAM II. The results showed consistent diagnostic accuracy across these three lots, but further lot-to-lot variability may exist, as observed in a previous study of six lots of FujiLAM. To fully capture potential variability, further studies, including a larger number of lots, would be beneficial to ensure the reliability of FujiLAM II across broader production runs. Second, the diagnostic accuracy of FujiLAM II for eMRS and CRS could not be assessed because only MRS was used as the TB diagnostic criterion. Finally, urinary LAM concentrations were not measured. Consequently, it remains unclear whether urine specimens contain LAM concentrations near the detection limit of FujiLAM II.

In conclusion, the FujiLAM II demonstrated no lot-to-lot variability and exhibited high sensitivity and specificity for TB diagnosis in patients with CD4 cell counts <200 cells/µL. The FujiLAM II is a highly accurate point-of-care test for TB diagnosis in PLHIV.
